# Everyday music listening and affect regulation: The role of MP3 players

**DOI:** 10.3402/qhw.v8i0.20595

**Published:** 2013-08-07

**Authors:** Marie Strand Skånland

**Affiliations:** Department of Musicology, University of Oslo, Norway

**Keywords:** Mobile music, music listening, self-regulation, emotions, emotional intelligence, subjective well-being

## Abstract

The use of digital portable music devices such as MP3 players has rapidly increased during the last decade, and the sheer *availability* of music offered by such players raises questions about their impact on listeners’ mental and physical health and well-being. This article explores MP3 player use as an everyday tactic for affect regulation, here understood as an individual's efforts to maintain or change the intensity or duration of a given affect. The ability to understand and regulate affects has significant health implications, and among the tactics relevant to such regulation, engagement with music has proven to be particularly successful. The material presented in this article is based on a qualitative interview study focused on MP3 player use as a medium for musical self-care. Because MP3 users can listen to whatever they want, whenever they want, and target their music in the interests of managing and regulating moods and emotions, the MP3 player represents a valuable and convenient technology of affect regulation.

The MP3 player has become a principal medium for everyday listening to music, because users can carry with them a vast amount of personally selected music wherever they go. The global MP3 market reached 225 million players in 2009 (InStat, 2009), and by 2011, Apple—which holds a 70% market share—had sold 300 million iPods worldwide.^1^ In Norway, over 50% of the general population and over 70% of the youth population (16–24 years old) used MP3 players daily in 2011 (Vaage, 2012). This unprecedented availability of music to people and the accompanying widespread use of portable music players raises questions about their actual impacts on the user. Research into the active use of MP3 players is therefore necessary to develop an understanding of how mobile music listening might influence the listener and, in turn, how it might be used as a resource for self-regulation.

Vohs and Baumeister define self-regulation as “any efforts by the human self to alter any of its own inner states or responses” (2004, p. 2). Affect regulation is one important aspect of self-regulation that has proved vital to a sense of well-being, positive mental health, and effective human functioning (Fave, 2006; Grewal & Salovey, 2006; Gross & Muños, 1995; Larsen, 2000; Larsen & Prizmic, 2004). Music, in turn, is one of a number of tactics people use for affect regulation (Thayer, Newman, & McClain, 1994; Van Goethem & Sloboda, 2008), and it is particularly efficient in this regard (Saarikallio & Erkkilä, 2007; Thayer et al., 1994; van Goethem, 2010). In what follows, I will engage with the ways in which people practice affect regulation in their everyday lives by listening to music. Among others, DeNora (1999, 2000) has shown that people are extremely adept at using music in their daily lives as a form of “caring for the self”. My approach to this phenomenon acknowledges the fact that individuals use music (in everyday life) as a resource in their self-care and thereby encompasses those individuals’ strengths and competencies.

This article explores the research question of how the MP3 player may be used as a resource in affect regulation. The MP3 player accommodates personal music choices in any context and performs the specific function of creating an individual space for listeners so that they may focus on their own emotional states. This is an important characteristic of the MP3 player, in that listeners are able to attend to, manage, and regulate themselves via listening to music both inside *and* outside the home.

To understand how (mobile) music listening might contribute to affect regulation, we must first understand what affect regulation is, and what role it has in well-being and health.

## Affect regulation, well-being and health

Affect is commonly used as an umbrella term for emotions, mood, stress responses, and emotional episodes (Gross, 1999). In the present text, I will focus in particular on mood and emotions.

Though it can be difficult to distinguish between emotions and mood, the former may be defined as relatively short and intense reactions to goal-oriented changes in the environment (Juslin, 2009; Juslin & Laukka, 2004). Moods usually last longer than emotions and are less intense. In addition, moods are not oriented towards a fixed object or incident, whereas emotions mobilize the body in a reaction linked to a specific phenomenon. While our moods inform us about our inner states, our emotions inform us about the environment and external incidents (Juslin & Laukka, 2004; Larsen, 2000). (For more on shared features and differences between moods and emotions, see Gross, 1998; Larsen, 2000.)

Larsen and Prizmic (2004) note that the several definitions of affect regulation share an acknowledgement that “in the process of monitoring and evaluating affective states, individuals take action either to maintain or to change (enhance or suppress) the intensity of affect, or to prolonged [*sic*] or shorten the affective episode” (2004, p. 40). Moods and emotions often contain important information for the individual who experiences them. It is necessary to feel both joy and sorrow in order to learn and to evolve, and affects are a vital source of this kind of feedback. However, affective states can sometimes persist long after their function has been fulfilled (that is, the feedback has been perceived) and consequently become dysfunctional. “The ability to self-regulate affective states – the ability to hang up after getting the message – is thus a crucial part of effective and adaptive psychological functioning”, Larsen notes (2000, p. 129).

Gross and Muños (1995), among others, have argued that the ability to regulate affect is a vital aspect of mental health and, more recently, Larsen and Prizmic (2004) claimed that the ineffective regulation of negative affect is probably a significant factor in depression and mood disorders. This view is supported by Grewal and Salovey (2006), who posit a relationship between the ability to manage one's emotions (or “emotional intelligence”, defined as the ability to perceive, understand, and manage emotions) and mental health. Put differently, there seems to be a relationship between low emotional intelligence and some forms of mental illness (Grewal & Salovey, 2006).

Gross and Muños further state that emotion regulation is vital to adult function and fundamental to all situations, solitary or collective, public or private: “When emotions become dysregulated”, they write, “it is clear that something is very wrong” (Gross & Muños, 1995, p. 155). Moreover, they point out that while emotion regulation in adulthood is so pervasive that it seems to be taken for granted, we must not overlook its importance to general mental health (Gross & Muños, 1995).

Health, of course, is in itself a challenging and varied concept. In contrast to the biomedical model of health, which is mainly concerned with the absence of disease, I am interested here in the social model of health, which understands health as a positive state of wholeness and well-being, or as the experience of vigour and a surplus of energy. It is not unusual, after all, for people with disabilities to describe their health as “excellent” (Blaxter, 2004; Fugelli & Ingstad, 2009), reflecting a notion of health related to the experience of quality of life, defined by the World Health Organization thus:

An individual's perception of their position in life, in the context of the culture and value systems in which they live, and in relation to their goals, expectations, standards, and concerns. It is a broad ranging concept, affected in a complex way by the person's physical health, psychological state, level of independence, social relationships, and their relationship to salient features of their environment. (WHOQOL group, 1995, p. 1404, in Camfield & Skevington, 2008, p. 765)

Camfield and Skevington (2008) note that this definition of quality of life—which has been widely accepted—converges strongly with definitions of subjective well-being. Næss (2001) likewise defines quality of life as mental well-being—that is, an individual's conscious, *positive*, cognitive and mental experiences. This understanding is virtually equivalent to subjective well-being, which is characterized by a balance between pleasant and unpleasant experiences (Haybron, 2008; Larsen & Prizmic, 2008).

Affect regulation is closely related to subjective well-being (from now on simply referred to as well-being), because the latter can generally be regulated by increasing positive affect or decreasing negative affect (Larsen & Prizmic, 2008). Together with life satisfaction, positive affect and negative affect comprise the three-fold structure of well-being:

Thus a person is said to have high SWB [subjective well-being] if she or he experiences life satisfaction and frequent joy, and only infrequently experiences unpleasant emotions such as sadness or anger. Contrariwise, a person is said to have low SWB if she or he is dissatisfied with life, experiences little joy and affection and frequently feels negative emotions such as anger or anxiety. (Diener, Suh, & Oishi, 1997, p. 25)

Affect regulation is thus a central part of well-being and positive mental health. Several different strategies and tactics can be used to practice it, including, as we will see below, music listening.

## Research on music listening and affect regulation

People use a variety of different strategies and tactics for mood regulation, including working out, eating, calling a friend, taking a shower, watching TV, or shopping (Thayer et al., 1994). In Thayer and colleagues’ study of mood regulation, music represented a remarkably, and unexpected, successful tactic.^2^ Participants in their study often used music as a means of mood regulation, specifically to change a bad mood, increase energy level or reduce tension. These findings have been echoed in studies focusing on music and affect regulation as well (Saarikallio, 2007; Saarikallio & Erkkilä, 2007; van Goethem, 2010).

During the last decade, there has been a growing interest in the relationship between music listening and affects (Gabrielsson, 2010; Juslin, 2009; Juslin & Laukka, 2004; Juslin, Liljeström, Västfjäll, Barradas & Silva, 2008; Juslin, Liljeström, Västfjäll & Lundquist, 2010; Juslin & Sloboda, 2010; Saarikallio & Erkkilä, 2007; Sloboda, 2005a, 2005b, 2010; van Goethem, 2010; Västfjäll, Juslin & Hartig, 2012; Vist 2009). Saarikallio (2007) and van Goethem (2010) in particular have done important work towards documenting individuals’ use of music in their affect regulation. van Goethem has found music listening to be one of the most common tactics used for affect regulation and identifies six explanations for why this might be so:

1. Music is viewed as a quick and easy accessible “fix”.
2. Listening to music does not require any brainpower.
3. Music listening is easy to combine with other activities (and tactics).
4. Music listening allows a temporary break without leaving everything behind.
5. Music listening is healthier than other tactics, such as eating or smoking.
6. Prior experience leads to knowledge of possible outcomes. (van Goethem, 2010, p. 273)

It appears, among other things, that the easy availability of music makes it attractive as an affect regulation tactic. MacDonald and colleagues also note that music's ubiquity may help to explain some of its importance in the contexts of well-being and health (MacDonald, Kreutz, & Mitchell, 2012). With the MP3 player, in turn, the accessibility of *personally chosen* music is higher than ever, and it therefore appears relevant to look into the role of this particular technology in affect regulation.

Juslin and Laukka (2004) believe that, because people are generally able to choose what music they listen to, they favour music that makes them “feel good”. Larsen (2000), p. 131) also assumes that people in principle “do things for the sake of feeling good”. However, according to Erber and Erber (2000), this is an oversimplification at best—in fact, they challenge the rather hedonistic notion that “humans, by and large, seek pleasure and avoid pain” (Erber & Erber, 2000, p. 142). Instead, they believe that *context* is decisive for whether one chooses to maintain or dispose of either good or bad affects. They explain that we may well choose to indulge in negative affects when we are alone, whereas we are otherwise forced to regulate our moods and emotions according to the demands of the situation. I will return to this possibility below. However, before presenting findings from my research, I will outline the methods I employed.

## Methods

In this study, I sought to understand how individuals might utilize their MP3 players as a means of daily self-care, to maintain or promote their subjective well-being and mental health (Skånland, 2012). Qualitative interviews were chosen as method because I wanted to reach the subjective experiences of MP3 users. I was not just interested in people's habits and practices related to their MP3 use, in which methods such as the Experience Sampling Method (Sloboda, O'Neill, & Ivaldi, 2001), questionnaires, or observations could have led to valuable insights. Instead, I was interested in individuals’ *reflections* concerning their use of MP3 players and the subjective *meanings* of this use. As Kvale simply asks, “If you want to know how people understand their world and their life, why not talk with them?” (Kvale, 1996, p. 1). By carrying out in-depth interviews, the wish was to come close to the everyday life experiences of MP3 users and the meaning these experiences had for the informants (Flick, 2006; Kvale, 1996). Flick argues for use of qualitative methods as a way of reaching the subjective meaning of everyday experiences, and I considered qualitative interviews an appropriate approach to the study of the meaning of mobile music listening in everyday life.

The research presented here is based on interviews with six men and six women between the ages of 18 and 44, all of whom were Norwegian and lived in Oslo or the surrounding areas. They were all apparently healthy and able to function well in the world. The choice to study adult MP3 users was based on the fact that there have already been conducted several studies relating to teenagers and music (Laiho, 2004; North, Hargreaves, & O'Neill, 2000; Saarikallio & Erkkilä, 2007; Skarpeid, 2008; Tarrant, North, & Hargreaves, 2002; Wells & Hakanen, 1991). Although teenagers make up an important group of MP3 users, they are not alone in this use, as my sample shows. I therefore believe it is important not to restrict research to this group and have accordingly chosen to focus on adults.

The only condition for participation in this study was that informants used their MP3 players regularly. To recruit people, I posted information about the study at different locations in Oslo and circulated it via email to acquaintances, asking them, in turn, to forward the information to their own contacts. One informant contacted me after reading about the project in an interview with me in the Norwegian media, three contacted me after seeing a poster, and eight contacted me after being referred by a mutual friend. All hailed from a Western, urban culture and could generally be described as wealthy and well educated.

The interviews covered the ways in which the informants used music on their MP3 players in relation to various cognitive, emotional, and bodily aspects of their daily lives; we also discussed the informants’ experiences of their environments, personal boundaries, and social and private spaces when listening to their private music. The interviews were semi-structured and exploratory, in the sense that I was able to improvise over the course of the conversations in order to follow up on interesting leads. Still, the interviews were based on an interview guide with over 20 questions concerning the informants’ experiences with their MP3 players. The guide functioned as a framework for the conversations, and all of the informants were asked more or less the same sorts of questions: Why did they choose to use their MP3 players, and in what situations did they normally use them? Did they feel that this music listening affected them in any way? How did they choose music, how much did they listen to it, and how did they feel about the music? How did they use MP3 players in different situations: on a good day, when they were tired, on the way to a party, and so on? And how did private music listening affect their experiences of their environments and other people? Finally, I asked the informants whether they had any negative experiences using their MP3 players, and I asked them to reflect upon their daily lives in the absence of the player. The interviews were conducted in Norwegian, and a professional translated the quotations included here.

The interviews, which lasted about an hour apiece, were transcribed and coded, inspired by Kvale (1996), Smith (1995) and Giorgi (1985). In the first round of breaking down the data into meaningful units (i.e., *open coding*; Boeije, 2010), not much emphasis was put on the relevance of the research material, since it was still too early to know what would be of value and what would be irrelevant. I followed Smith's course of action, gathering all occurrences of a theme in one category. In the next round (i.e., *axial coding* (Boeije, 2010)), theory was merged with the data. This brought out new understandings of the phenomenon and led to some adjustments of the categories. In the end, the data were divided into three main categories: “use of the MP3 player” (with subthemes such as routines, choice of music, listening outdoors versus indoors, and the importance of the MP3 player), “self-regulation” (including affect regulation, cognitive regulation, and bodily regulation) and “coping” (including boundaries, sense of control, and negotiating the urban environment).^3^ The present article focuses on one of the subthemes, namely that of affect regulation, which was by far the most frequent to appear in the data material.

Because conducting qualitative interviews is time-consuming, it limits the number of informants I could include in the study. This means that the research could not result in general laws. Nevertheless, the method was chosen because I was more interested in subjective experiences rather than universal knowledge. Results from one research-context cannot be automatically transferred to a different context (Holt, 1991), but I will nonetheless say something here about the context of the present research and possible transferability to other contexts.

First of all, the study was carried out in a Western society. The informants lived or worked in Oslo, which places this study in an urban context. Carrying out a similar study in a different environment could possibly produce different findings. For example, the informants valued the possibility of creating private spaces with their MP3 players, which might be a characteristic of the city, where people tend to keep to themselves in spite of, or because of, the large amount of people. Second, the informants were adult. Presumably, teenagers and children use music in different ways. We know that teenagers experience more intense emotions than adults do (Laiho, 2004), and a study by Bergh and colleagues on adolescents’ engagement with MP3 players (Bergh, DeNora, & Bergh, in press) implies that these young listeners might incorporate their MP3 players in social interaction to a larger extent than the informants in the present study. Next, the informants are educated. This says something about social class, and it is not given that people from different social sets use MP3 players in the same ways. Also, economy may lead to limitations when it comes to transferability of the findings, both when it comes to the ability to buy an MP3 player, and when it comes to differences in functionality of the players. That being said, the informants in this study owned players of varying functionality and capacities. Finally, the informants are presumably healthy and well-functioning. They are seemingly capable of self-regulation and might therefore use music as a resource in affect regulation in different ways from people who struggle with affect regulation or other health issues.

Given these restrictions concerning the context of the study, I do nonetheless believe the findings are valid for a larger group of people. First of all, I believe people who use MP3 players in general will relate to some of the findings, whether they are young, elderly, healthy, ill, educated, uneducated, from the countryside, or the city. Further, I believe people who listen to music in general will identify with some of the findings. This study relates to other studies with similar findings, namely that people use music according to personal needs (Bull, 2000, 2007; DeNora, 2000; Greasley, 2008; Juslin & Laukka, 2004; North, Hargreaves, & Hargreaves, 2004; Sloboda, 2005a, b; Sloboda et al., 2001). Hence, *music listening* becomes the link.

As mentioned above, this article focuses on the subtheme of affect regulation. While I believe that cognitive, emotional and bodily states are in fact closely intertwined and only brook distinction on a theoretical level, I nevertheless find it useful to focus on affect regulation alone here, given the framework of this article. In the next section, I will present some of the empirical data related to this topic.

## The MP3 player: a technology of affect regulation

Although it can be difficult to differentiate between moods and emotions, I have nevertheless chosen to divide my findings into the categories of “mood regulation” and “emotion regulation” in an attempt to structure the material. The informants generally spoke of moods and emotions interchangeably, so this distinction is based on my interpretations of the interview data and informed by theories on affect regulation, mood, and emotions, as presented above (Erber & Erber, 2000; Gross, 1998, 1999; Gross & Muños, 1995; Larsen, 2000; Larsen & Prizmic, 2004, 2008; Thayer et al., 1994). Let me begin by exploring the relationship between mobile music listening and mood regulation.

### Mobile music listening and mood regulation

Mood was a recurrent theme in all of the interviews that I conducted. Evidently, music and mood are closely connected, and mood was always a factor when the informants listened to their MP3 player. With regard to her MP3 player, one of the informants said, “I know what to do to get into certain moods”, then baldly stated, “I use [music] to get into the mood I want to be in” (female, 27). Others applauded the efficiency of MP3 music over other regulating tactics. One woman tried to envision her daily life without her MP3 player:

If I didn't have the MP3 player, I could of course have music at home or somewhere, but the choice to listen to the music *I* like wouldn't be there. And then I had to find other methods of getting into different moods, which isn't easy. It's *very* effective with music, so there's a great difference between using music to get into a mood and using other means. (Female, 37)

This informant stressed that the MP3 player offered her more freedom of choice over what music to listen to than other listening devices did. The opportunity to choose from a large, private music collection—one that is always available—is apparently a leading recommendation for the MP3 player over other regulation devices. Another woman described the value of being able to regulate her mood after work and *before* she arrived home to husband and children, and she credited her MP3 player for providing her with this opportunity.

In addition to the sheer availability of the music on it, an important aspect of the MP3 player was that it created a private space within which the informants could more easily focus on their own states of mind. This enabled them to control their moods without being influenced by their surroundings (or other people). One informant described how this worked:

Often, one can be somewhere where … there is a kind of atmosphere in the room, either because there are people there or there's something with the place [itself]. One can then use the music … to take a bit of control, [to] create one's own little private room.

*Do you find that you can pull back from your surroundings with the music, or how does this work?*

Yes, I think so, too. Absolutely. Yes, it's a little like that, … you simply separate yourself from your surroundings … when it comes to thoughts and moods and feelings. [It is] a way to isolate yourself. (Female, 26)

This informant used MP3 music to manage her state of mind precisely by isolating herself from her surroundings, adding, “At least I can control more [about] the influence my surroundings have on me by having my MP3 player, because then I can decide myself, a little, the mood with which I want to meet the world.” She described her music as an “armour” against outside stimuli, characterizing it as vital not only to creating boundaries between her and her surroundings but also to filling the “private room” that results with a desired mood or emotion. Hence, music both created boundaries around a private space and added contents to it at the same time. With the MP3 player, then, she could shut the door in her head and focus on herself rather than those around her.

While private music was used to induce, prolong, enhance, or change moods, the informants did not always have a desired mood, or so-called set point (see Larsen, 2000), towards which they aimed. Rather, they seemed to determine the mood they wanted by searching through music for what “felt right”, then using it to induce or enhance the mood in question. Finding the “right” song was very important to the informants. Even if they had several gigabytes of music in many genres on their MP3 players, they were typically very particular about the “right” and “wrong” type of music for any occasion. Though they did not always know what they wanted to listen to before they pressed play, they were very aware of what they did *not* want to listen to. Finding the right music could be a challenge:

Sometimes, it's a little difficult, because, in a way, I have to find the feeling—that one, [that] ‘Oh, that feels right—no, maybe it's the other one’ [laughs]. So sometimes I go through all of the albums, and then I think, ‘This can't be such a big deal, I can just choose a track’. But sometimes I feel it's right in a certain situation but it's a different type of situation [now]. And I've wondered: Why is it like that? ‘No, I feel that it's right to listen to a little rhythm—no, now I have to have something slow, or now I have to listen to some folk music, or classical, maybe?’ And it simply depends on my mood. (Female, 43)

Searching their music libraries for what “felt right” also helped to clarify the informants’ moods. Finding the “correct” song made it more apparent what mood they were actually in, and the music could help to illuminate, intensify and prolong this mood. Most of the informants said that they listened to music that matched the mood they were already in, and several of them explained that they preferred to stay in their current state rather than change it:

I like to be, for a little [while], in that mood that I'm already in and use it … I think my mood has something to offer me, so I have to just try and use it for what it's worth. (Male, 27)

While the informants normally listened to music that reflected or heightened their current moods, some also reported using music to effect a change of mood in situations where they considered such a change to be necessary or desirable. This agrees with the findings of DeNora's (2000) study, which showed that participants listened to music as a way to prepare for (or “get into the mood of”) social gatherings. The youngest informant explained:

If I'm going to a party or something, or if something lousy has happened and I've to go to school, for example, or to a social gathering, or just to a friend's or something, I put on some pleasant music to get in a better mood. (Female, 18)

But the informants also chose to change their moods simply according to their personal needs or preferences at the time:

I can also use [music] consciously …. For example, I can be annoyed or upset about something … and use the music actively to regain my balance, so that I don't waste so much energy and effort on things that don't work …. I can put on the iPod … and manage to relate to people in a different way. (Female, 37)

This is a good example of how music can be used to rid oneself of negative or destructive moods, such as annoyance. MP3 music made this woman feel more harmonious; she said it “strengthened her internal state of calmness” and made her more tolerant towards her surroundings and her work environment. She explained that when she realized that she could use her private music as a tool for regulating her moods, she experienced heightened control in a variety of other situations as well. As a regulation device, the MP3 player functions as a technology of internal control as well, which in turn empowers the listeners as active agents (Skånland, 2012).

### Everyday music listening and emotion regulation

Juslin and Sloboda (2001) point out that there is a fundamental relationship between music and affects, and the present research demonstrates it. One informant concluded, “In any case, I think music can always bring out one or another emotion in me”, asserting further that her emotions always played a part in her choice of music (female, 24). Another informant (male, 26) reported that he could control, intensify, or otherwise influence almost any emotion using music. In general, the informants looked to music to sustain or enhance their emotions, and thereby to take control of them.

Interestingly, several of the informants said that they became more positively attuned to their environments when they listened to their MP3 players. One informant (male, 27) thought he came across as more affirmative, open, and “contact-seeking” when he listened to MP3 music. Another informant (female, 18) said flatly that the music she listened to defined how she perceived and responded to her surroundings—some music brought about a more positive attitude, while other music did the opposite. She continued, “If I'm listening to music, then a lot of chaos in town will be just fantastic, and there are only masses of fine people when you are listening to happy music”. Music she enjoys allowed her to cope better with what might otherwise be perceived as stressful surroundings. It also enabled her to interact with others in a more positive way: “If I'm listening to good music, I go around smiling a little to everyone, and almost go and dance a little for myself. I think it's really fun.” A number of the other informants also mentioned that their attitude towards their surroundings and their perceptions of other people became more positive when they were listening to music.

On good days, of course, the informants naturally listened to music that sustained or enhanced their existing affect. One noted that he could.

shut out bad feelings, because it becomes more difficult for these to take hold, as it is harder for difficult thoughts to take root when I'm listening to music that makes me happy (male, 26).

In other words, music seems to fill a prophylactic function against unwanted thoughts and emotions. The informants often described music listening as pleasurable, and one added that music can “cheer you up” (male, 43). However, the informants in this study usually listened to music to *prolong* or *intensify* happy feelings rather than to *create* positive emotions when they were feeling low.

Vist (2009) observes that it is somewhat simplistic to divide emotions into “positive” and “negative” groups, as I have done here, noting that music can also “beautify the sorrow” and otherwise make the bad seem good for a time. One informant seemed to endorse this perspective when I asked her how she felt after listening to sad music:

I don't quite know if I'm able to tell whether I become sadder or happier. But it may be that [it helps], perhaps, to connect the sadness, which is a lousy feeling in itself, to something bigger. For things are often beautiful when they are sad, kind of melancholic, [so] that one in fact links the sadness to something positive. As such, I think I can experience [the fact] that by listening to sad music when I'm sad, I don't necessarily become sadder but just link the sadness to something else. (Female, 26)

This woman used music to connect her sadness, a “lousy feeling”, to something positive, something outside of herself, which made it easier to bear. Relating to the music also made her feel less alone in what she was experiencing, and more capable of dealing with it:

I think at least I can control it, and the knowledge that there are certain songs or certain artists that make me sad, because I have some sad experiences I've had while I listened to that music, or only because it's the music that has, in a way, a sadness in it.

This evokes another observation rooted in DeNora's (2000) research, whereby participants were seen to act as their own personal disc jockeys. Informants’ prior experience with music, in other words, determines much about their use of it as an emotion-regulating tactic.

By browsing through the music library on the MP3 player for music that “feels right”, informants gained insight into their states of mind, whether positive or negative. When they found music that matched their current affects, they found those affects to be clarified as well:

Sometimes I can use [music] to investigate [my mood] a little. [It's] good to go around and just listen to music, listen to something that perhaps allows reflection …. In a way, it can help me to find out what mood I'm in, and to feel it …. And then maybe manage to understand why, and to do something about it (Male, 24).

I actually use music to amplify the mood I'm in already …. [The mood] becomes clearer. If I'm a bit angry, I put on some angry music and just feel the anger and let it flow, simmer …. [The music] helps me maybe get out of the pit …. Instead of just being angry, I'm able to distance myself from the feeling and monitor the feeling, in a way … examine the feeling, touch it and study it a little. (Male, 27)

In using music to reflect on and examine their emotions, the informants gained insight into those states of mind and a better general understanding of their affective lives. In turn, this may have helped the informants deal better with their emotions.

## Discussion

It is evident that the present study's informants used their MP3 players as resources in affect regulation, both consciously and subconsciously. Importantly, they did *not* necessarily aim to feel better by listening to music but rather to sustain and/or reflect upon whatever moods and emotions they were experiencing. Unless the situation appeared to demand it, the informants seldom tried to change their negative affects but rather tried to (temporarily) indulge in them. Thus, informants did not always choose music that increased their positive emotions, which conflicts with Juslin and Laukka's (2004) assumption that individuals generally tend towards music that makes them “feel good” or “feel better”. Positive emotions do dominate in relation to music in both Juslin and Laukka's (2004) and Sloboda and O'Neill's (2001) studies. However, the *listening context* generally determines which emotions the listeners will seek, as well as whether or not they will try to change or enhance those emotions with the help of music, in accordance with Erber and Erber's (2000) theory.

By maintaining so-called negative affects with the help of music, furthermore, informants here gained a better understanding of their internal states. Only then could the informants successfully begin to change their moods for the better. In van Goethem's (2010) study, interestingly, the participants who experienced the most successful affect regulation used a stepwise approach. Attempts to regulate affects all at once—for example, trying to change a negative mood by listening to “happy” music—were often unsuccessful.

We have seen that affects offer important information and feedback to the person experiencing them (Grewal & Salovey, 2006; Larsen & Prizmic, 2004). As Larsen and Prizmic point out, the goal of affect regulation is not to short-circuit all affects but rather to abbreviate the current affect after “receiving its message”. This is something that the informants in this study obviously understood. By listening to music that sustains their current mood, these people created an opportunity to come to terms with that mood and accept its feedback. While public environments and urban surroundings can be disturbing and distract from one's internal state, the MP3 player restores that focus and extends or abbreviates one's mood as need be.

The opportunity provided by the MP3 player to reflect on one's mood and emotions might lead to improved insight into one's affective life, which in turn might lead to enhanced emotional intelligence. Grewal and Salovey (2006), as noted above, claim that emotional intelligence plays a vital part in both physical and mental health. Moreover, they demonstrate a relationship between low emotional intelligence and some forms of mental illness, such as depression, alexithymia, and borderline personality disorder. The informants in this study were apparently in good mental health, and most of them demonstrated the ability to reflect on and manage their affects. They would therefore appear to be in little danger of developing mental or physical illnesses related to poor emotional intelligence. Nevertheless, if music listening were able to improve their ability to understand and manage their affects, it would have the potential of developing their emotional intelligence further as well. In this sense, music listening would represent a means of staying healthy, or even promoting positive mental health.

## Concluding remarks

It appears that the informants in this study all used the private and portable music on their MP3 players to regulate their affects. Via the MP3 player, of course, music is always available. Many MP3 players also have large capacities, which enable listeners to carry a vast amount of private music with them at all times. Hence, listeners are generally able to find music that agrees with their current or desired affects, enabling them in turn to manage and regulate their mood and emotions. The private listening device also permits listeners to carve out a private space even when they are in a public environment, which seems to be particularly desirable to individuals in urban environments. These private spaces allow the listeners to focus on their own state of being, enabling them to control and regulate their affects without interference from their surroundings.

As we have seen, affect regulation is important to well-being and health, and MP3 players and private music listening represent viable tactics for this form of self-regulation. It is apparent that MP3 players are indeed experienced as efficient technologies of affect regulation, and that van Goethem's six explanations for music's primacy in this regard (see above) are true. I have shown that the informants’ knowledge of how music works for them allowed them to use it as an efficient regulation tactic (explanation 6). Furthermore, the MP3 player acquired a particular role via the availability of music, allowing listeners to combine music listening with other activities when “on the go” (explanations 1, 3, and 4). Although I would argue that music does indeed require brainpower (explanation 2), it can also be used for relaxing and unwinding in a different manner than, for example, listening to the news. In sum, listening to music on MP3 players appears to be an easily available and efficient tactic for affect regulation—and, with the exception of hearing damage, without the physical side effects of other, more harmful regulation tactics such as smoking or drinking (explanation 5).

Of course, not all MP3 users are the same. They are individuals with different listening habits, different needs, and different experiences. However, comparing my findings to research done on personal stereo-use (Bull, 2000, 2007) and music listening in everyday life (DeNora, 2000; Greasley, 2008; Juslin & Laukka, 2004; North et al., 2004; Sloboda, 2005a, b; Sloboda et al., 2001) makes it apparent that there are obvious similarities in how music is used. There are also some distinct similarities in how the different informants in the present study experience their MP3 use. This leads to the assumption that the findings from the current study should be transferable to other contexts. Considering that the number of people using MP3 players on a daily basis in Norway was over 50% of the general population and over 70% of the youth population in 2011 (Vaage, 2012), the findings from the present study should be transferable to other urban MP3 users in Norway, and to other urban, Western societies as well.

## Footnotes

1 See http://en.wikipedia.org/wiki/IPod#cite_note-71

2 van Goethem (2010) has developed a framework for affect regulation where she distinguishes between affect regulation goals, strategies, tactics and mechanisms, and music listening is described specifically as an affect regulation *tactic*. I have chosen to use her framework.

3 For a more detailed description of the analytic process, see Skånland, 2012.
